# Aerobic exercise in severe mental illness: requirements from the perspective of sports medicine

**DOI:** 10.1007/s00406-021-01360-x

**Published:** 2021-12-06

**Authors:** Peter Falkai, Andrea Schmitt, Christian P. Rosenbeiger, Isabel Maurus, Lisa Hattenkofer, Alkomiet Hasan, Berend Malchow, Pascale Heim-Ohmayer, Martin Halle, Melanie Heitkamp

**Affiliations:** 1 Department of Psychiatry and Psychotherapy, University Hospital, LMU Munich, Nussbaumstrasse 7, 80336 Munich, Germany; 2 Laboratory of Neuroscience (LIM27), Institute of Psychiatry, University of São Paulo, São Paulo, Brazil; 3 Department of Psychiatry and Psychosomatics of the University Augsburg, Medical Faculty, Bezirkskrankenhaus Augsburg, University of Augsburg, Augsburg, Germany; 4 Department of Psychiatry and Psychotherapy, University Medical Center Göttingen, Von-Siebold-Str. 5, 37075 Göttingen, Germany; 5 Department of Prevention and Sports Medicine, Medical Faculty, Technical University of Munich, University Hospital ‘Klinikum Rechts der Isar’, Munich, Germany; 6 DZHK (German Center for Cardiovascular Research), Partner Site Munich Heart Alliance, Munich, Germany

**Keywords:** Major depression, Bipolar disorder, Schizophrenia, Aerobic exercise, Physical activity, Cardiorespiratory fitness, Neuroplasticity

## Abstract

Major depression, bipolar disorder, and schizophrenia are severe mental illnesses. Despite receiving psychopharmacological and psychosocial treatments, about half of patients develop a chronic course with residual cognitive and negative symptoms and have a high risk for cardiovascular disease and reduced life expectancy. Therefore, add-on innovative treatment approaches are needed to improve outcome. Aerobic exercise interventions have been shown to improve global functioning, cognition, and negative and depressive symptoms in these patients. The basic mechanism of these exercise-related changes has been reported to be improved brain plasticity, e.g., increased volume of disease-related brain regions such as the hippocampus. The optimal type, duration, and frequency of exercise have not yet been determined and need to be addressed in supervised physical exercise studies. Because of the low physical activity levels, lack of drive related to negative and depressive symptoms, and high prevalence of cardiovascular comorbidities in patients with severe mental illness, besides aiming to improve symptoms of mental illness, exercise interventions should also aim to increase cardiorespiratory fitness, which they should comprehensively assess by direct measurements of maximal oxygen uptake. Based on the recommendations for developing cardiorespiratory fitness by the American College of Sports Medicine, 150 min moderate-intensity training per week or vigorous-intensity exercise training for 75 min per week are appropriate. Most studies have had relatively short intervention periods, so future studies should focus on long-term adherence to exercise by implementing motivational strategies supported by telemedicine and by identifying and targeting typical barriers to exercise in this patient population.

## Introduction

In 2010, more than 25% of the population in Europe was diagnosed with a mental illness [1]. Among mental illnesses, major depression (MDD), bipolar disorder (BD) and schizophrenia (SZ) belong to the 20 most burdensome disorders and result in annual costs of 207 billion euro in Europe. Thus, besides cardiovascular diseases [2, 3], mental disorders—especially MDD—are one of the leading illness-related causes of years lived with disability worldwide [4]. Furthermore, more than 50% of patients in Europe with a mental illness, equivalent to about 25 million Europeans, develop a relapsing, chronic course of their illness with residual symptoms, which are associated with poor functional outcome [5].

Epidemiological research showed that in the last 100 years the long-term outcome of SZ has remained relatively stable [6], despite the introduction of psychotropic medications 70 years ago. Furthermore, psychotropic medications also have not improved outcome dimensions like cognition or negative symptoms. Overall, about 20% of patients with SZ have a good outcome and are able to participate in the primary job market, maintain a stable partnership and have longer phases with no need for psychopharmacological support [7, 8]; about 30% of patients will have a good prognosis but will need to take long-term psychotropic medication, which can have burdensome side effects, especially related to metabolic syndrome [9]; and about 50% of patients will develop a chronic course (defined as continuous symptoms over a period of at least 2 years) with different degrees of residual symptoms and disability [5]. Residual symptoms include cognitive dysfunction, impaired mood, reduced drive, and reduced ability to cope with stress. These individuals have an unfavorable long-term social outcome, meaning that they have difficulties finding a long-term job on the primary job market or maintaining a stable partnership [8].

Besides having a direct influence on functional outcome, environmental risk factors also affect mortality: The mortality rate from physical causes, including suicide is 20-fold higher in patients with unipolar depression than in the general population, 15-fold higher in patients with BD, and 12-fold higher in patients with SZ [10–13]. Another important cause is the high incidence of medical comorbidities due to unhealthy lifestyle habits, such as high rates of cigarette smoking and low levels of physical activity. Estimates indicate that together suicides and lifestyle-related factors reduce life expectancy by nearly 10 to 20 years compared with the general population [14, 15].

A meta-analysis of data from 29 countries on 6 continents confirmed that people with mental disorders have a significantly higher mortality rate and that, in 65 studies, the highest mortality rate (relative risk 2.54; 95% CI 2.35–2.75) was among patients with psychosis [15–18]. People with severe mental illness have a higher risk of developing coronary heart disease than controls (adjusted hazard ratio 1.54; 95% CI 1.30–1.82) and a higher rate of autonomic nervous system dysfunction, including diminished heart rate variability, hypertension, alterations of the QT interval, and lipid pattern abnormalities [19]. Unfortunately, the currently available psychopharmacological and psychosocial treatments do not ameliorate or improve these symptoms significantly and therefore do not help to improve functional outcome or increase life expectancy.

Surprisingly few studies have examined whether aerobic exercise combined with diet and psychosocial interventions can reduce the mortality gap between patients with mental disorders and the general population, and the studies included only small samples of patients [20]. The seminal paper by the Lancet Commission [21] identified an unhealthy diet and a sedentary lifestyle as major risk factors for the physical illness burden of patients with MDD, BD, and SZ, diseases that are also collectively referred to as affective and nonaffective psychoses. The paper gives recommendations to influence these modifiable risk factors, e.g., including regularly exercise in treatment programs.

This qualitative review will outline the effects of aerobic exercise on clinical outcome in patients with MDD, BD, and SZ and will evaluate the quality of intervention studies on the basis of the requirements of modern sports medicine.

### Physical activity, physical fitness, and medical health outcome in patients with MDD, BD, and SZ

Low physical activity levels and poor cardiorespiratory fitness (CRF) are associated with a high risk of cardiovascular disease and all-cause mortality [22]. Exercise and good CRF play an important role in mitigating cardiovascular disease risk factors, such as metabolic syndrome, which is defined as a combination of increased waist circumference; elevated fasting glucose, triglycerides, and low high-density lipoprotein cholesterol; and high blood pressure [23, 24]. A meta-analysis determined that the risk of metabolic syndrome was elevated in all patients with affective and nonaffective psychoses (32.6%, 95% CI 30.8–34.3%) and that the prevalence did not differ between patients with MDD, BD, or SZ [25].

Another meta-analysis found that patients with MDD had lower levels of physical activity (standardized mean differenc, − 30; 95% CI − 0.40 to 0.21) and higher levels of sedentary behavior (standardized mean difference 0.99; 95% CI 0.01–0.18) than healthy controls [26]. Therefore, researchers concluded that less physically active patients might benefit from specific aerobic exercise interventions aimed at increasing physical fitness [27]. An important aspect to consider in this context is that low physical activity is related to negative symptoms such as amotivation [28], so aerobic exercise interventions must be supervised by experienced sports scientists to ensure that patients adhere to the intervention [29].

Physical fitness and physical activity are low not only in patients with MDD, but also in those with BD [30]. However, this diagnostic group is highly underrepresented in physical activity studies.

A study in patients with SZ showed that low physical fitness was associated with a higher prevalence of metabolic syndrome and more severe cognitive, negative, and positive symptoms [27]. The exercise capacity (measured by the distance covered in the 6-min walking test) of patients with SZ and prediabetes was reduced and the body mass index was increased; in addition, patients with SZ and manifest type 2 diabetes were less physically active [31].

Previous aerobic exercise studies showed the feasibility of endurance training in patients with SZ, and adaptations to aerobic endurance training in patients were comparable to those in healthy controls, as assessed by physical working capacity and maximal achieved power. However, differences were detected in changes of performance at a lactate concentration of 3 mmol/L, i.e., patients with SZ showed an impaired increase in lactate [29].

Different types of interventions have been evaluated that aim to improve physical health in patients with mental illness. A meta-analysis of 47,231 patients with SZ summarized and compared the effects of pharmacological and nonpharmacological interventions [32]. The authors showed that the most effective interventions for weight reduction were individual lifestyle counseling and exercise interventions, followed by psychoeducation, augmentation with the atypical antipsychotic aripiprazole, topiramate add-on therapy, and dietary interventions. The best efficacy in reducing glucose levels was found for a switch from olanzapine (the atypical antipsychotic with the highest risk for metabolic syndrome) to aripiprazole and add-on medication with metformin. Efficacy was also shown for treatment with glucagon-like peptide-1-receptor agonists, dietary interventions, and aripiprazole augmentation. Insulin resistance improved best followed by metformin treatment. Metformin also had the greatest effects on total cholesterol and high-density lipoprotein cholesterol. The best effect on triglycerides and low-density lipoprotein cholesterol was achieved with topiramate. Importantly, only exercise interventions increase exercise capacity [32]. Recent efforts to increase the efficacy of exercise include the use of high-intensity interval training (HIIT). In a randomized controlled HIIT study, compliant patients with overweight and SZ showed improvement in waist circumference, negative symptoms, and psychosocial functioning [33]. HIIT may be a feasible and effective way to improve CRF and metabolic parameters and has been established as such in physical disorders. It may also have more beneficial effects on the metabolic state than more moderate and continuous endurance training methods [20].

### Effects of aerobic exercise on symptoms of MDD

Aerobic exercise, often revered to as “endurance exercise”, is defined as physical activity with a predominant metabolic pathway that uses oxygen to meet energy demands (oxidative phosphorylation) and leads to only low blood lactate levels [34]. In practice, aerobic exercise is usually characterized by repeated sequences of physical activity in a light to moderate intensity for extended periods of time. Aerobic exercise improves especially CRF and includes typically activities such as walking, swimming or cycling [35]. Contrastingly, anaerobic exercise refers to short-term high-intensity efforts with a preponderance of metabolic pathways not using oxygen (phosphagens metabolic pathway and glycolytic pathway) [36]. In most studies, the term "anaerobic training" is used to describe high-intensity exercise intervals with a duration of up to several minutes that result in increased lactate levels.

Aerobic exercise studies addressing MDD are summarized in Table 1. An aerobic exercise training study of 12 × 75-min sessions over a period of 4 weeks in patients with SZ and MDD revealed improvements in cognition, which were more pronounced in the patients with SZ; however, the patients with MDD showed a greater reduction in depressive symptoms and anxiety [37]. Aerobic exercise studies in MDD showed that exercise improves working memory and psychosocial functioning and reduces depressive symptoms [38, 39]. In particular, one study provided evidence for an effect of aerobic exercise on remission in MDD by showing that 29.5% of patients with unremitted MDD remitted after 3 months of aerobic exercise treatment [40].

**Table 1:** Aerobic exercise studies in major depressive disorder, including study design, methodology, and clinical outcome

Year	Publication	Study design and diagnosis	Sample size, *n*		Intervention	Control	Measurement of CRF and PA	Intervention duration /follow-up	Outcome
			IG	CG					
2019	Asthon et al. 2020 [52]	Exploratory subanalysis of an RCT (145 of 181 who reported data on PA) Bipolar depression	(A) 50 (B) 46	49	(A) *N*-acetylcysteine alone (B) *N*-acetylcysteine with a combination of nutraceuticals	Placebo	MADRS at week 16 IPAQ-SF at week 4 MADRS at week 16 And others	16 weeks	PA was unrelated to change in depression symptoms across study duration In patients receiving combination treatment, total PA significantly predicted changes in bipolar depression symptoms
2019	Gujral et al. 2019 [97]	RCT, double blind Pilot study Major depressive episode	7	8	Venlafaxine XR AND supervised exercise Sessions: individualized, 3x/week, 1 h Moderate intensity (60–75% of age-based HR for ~ 45 min on a treadmill and/or cycle ergometer) Supervised: yes	Venlafaxine XR	CRF: submaximal *V*O2 test PA: Body Media Sensewear armband, triaxial accelerometer	12 weeks	No significant changes in fitness in the exercise group Significant reduction of depressive symptoms in both groups Association between improvement in fitness and increased cortical thickness in the anterior cingulate cortex
2018	Gerber et al. 2019 [98]	Secondary analysis of RCT MDD	53 Randomization: n.a		(A) Sprint interval training: 25 repetitions of 30 s high-intensity burst at 80% of max. power output, followed by 30 s of total rest (B) Continuous aerobic exercise training: 20 min continuous aerobic exercise on a bicycle ergometer with an intensity level of 60% of the maximal power output Both sessions: 3*x*/week, 35 min Intensity: prescribed individually to each participant Supervised by an experienced exercise coach	n.a	CRF: *V*O2_max_, bicycle ergometer Fitness Questionnaire, not specified	4 weeks	Improvements in *V*O2_max_ were associated with fewer depressive symptoms, better mental wellbeing, and better sleep post-intervention Improvements in perceived fitness were associated with less depression symptoms and better sleep higher mental wellbeing post-intervention Improvements in *V*O2_max_ and perceived fitness were associated with favorable changes in depressive symptoms, mental wellbeing, and sleep
2018	Minghetti et al. 2018 [48]	RCT MDD	(A) 30 (B) 29		(A) Continuous aerobic exercise training [108]: 20 min continuous exercise at a power output corresponding to 60% of the maximal power output (B) Sprint interval training (SIT): 25 repetitions of 30 s high-intensity bursts at 80% of maximal power output followed by 30 s of total rest (remaining seated on the bicycle) Sessions: 3*x*/week, 35 min Supervised by an experienced exercise coach Medication was counterbalanced in both intervention arms	n.a	CRF: exhausting incremental exercise test, bicycle ergometer Beck Depression Inventory-II	4 weeks	BDI-II scores substantially decreased in both groups, while submaximal and maximal variables improved in both groups Short-term SIT leads to similar results as CAT in patients with MDD
2018	Patten et al. 2019 [44]	Pilot Study Depressive symptoms	18	18	Free membership in fitness center for 12 weeks AND Six 30-min individually tailored sessions with an exercise counselor; included aerobic exercise, strength training, stretching, and recommendations to exercise regularly Exercise at the fitness center and at home were both encouraged, but no supervised exercise was provided	Free membership in fitness center for 12 weeks but no additional intervention	CRF: 6–12-min submaximal cardiorespiratory test, cycle ergometer PA: evaluation of trunk flexibility, resting heart rate, blood pressure and body composition measured by bioelectrical impedance Questionnaires: Stage of change for exercise, IPAQ, Beck Depression Inventory-II	12 weeks	No group differences were found in IPAQ or BDI-II scores at week 12. Increases from baseline in IPAQ moderate/vigorous activity minutes were associated with decreases in BDI-II scores at week 12
2017	Rethorst et al. 2017 [40]	Randomized, secondary analysis MDD			Two exercise doses: (A) 4 kcal/kg/week (B) 16 kcal/kg/week Exercise intensity was self-selected and monitored with an HR monitor Supervised: yes (complete dose in week 1, two in week 2, and 1 in week 3–12; rest unsupervised)			12 weeks	Predictors of remission were higher levels of brain-derived neurotrophic factor (BDNF) and Interleukin-1B, greater depressive symptom severity, and higher post-exercise positive affect. Predictors of treatment non-response were low cardiorespiratory fitness, lower levels of IL-6 and BDNF, and lower post-exercise positive affect. Models including these predictors resulted in predictive values greater than 70% (true predicted remitters/all predicted remitters) with specificities greater than 25% (true predicted remitters/all remitters)
2015	Carneiro et al. 2015 [41]	RCT; only women Clinical depression	13	13	Aerobic exercise group: indoor/outdoor natural circuit workouts AND Pharmacotherapy Sessions: 45–50 min/week; 3*x*/week Intensity: based on baseline fitness. First month: at least 65% of %HRmax; second month: to 70%; third month: 80%. Diverse Supervised: yes Motivational strategies (e.g., multidisciplinary teams; Facebook page; outings in the sunlight and in pleasant settings; etc.)	Pharmacotherapy only	Physical functioning: Distance walked in 6 min Number of times they could sit and stand from a chair in 30 s A seated medicine ball throw	4 months	Decrease in BDI-II and DASS-21 total score scales in exercise group. Relative to DASS-21, a significant decrease in anxiety and stress is found Improvement in relation to physical functioning parameters in exercise group Anthropometric parameters only significant different between groups in fat mass percentage No differences between groups in weight, body mass index, waist circumference, and self-esteem
2015	Doose et al. 2015 [42]	RCT Unipolar depression	30	16	Walking/running aerobic exercise program at a local sports club Sessions 3*x*/week, 60 min, outside Intensity: self-selected exercise intensity according to perceived exertion Supervised by teams of coaches and medical students	Wait list	CRF: Fitness Index, *V*O2_max_ as estimated or UKK 2 km Walk Test	8 weeks	Large reduction of depressive symptoms in HRSD-17 scores. BDI-II, FI scores, and VO2 max did not change significantly
2015	Kerling et al. 2015 [92]	Randomized Pilot trial Inpatient Moderate to severe depression	22	20	Exercise training 3*x*/week, 45 min: 25 min bicycle and 20 min cross trainer, stepper, arm ergometer, treadmill, etc. as preferred Moderate intensity: 50% of maximum workload from incremental test; above the VAT and below anaerobic lactate threshold Supervised by physicians, group format	Treatment as usual	CRF: *V*O2_peak_, VAT, Watts, lactate on bicycle ergometer MetS	6 weeks	Cardiorespiratory fitness (*V*O2_peak_, VAT, Watts), waist circumference and HDL cholesterol significantly improved in exercise group. Treatment response (expressed as ≥ 50% MADRS reduction) was more frequent in the exercise group
2014	Danielsson et al. 2014 [47]	RCT MDD			(A) Aerobic exercise: Training in the rehab center (e.g., cross trainer, jumping ropes, stationary bikes, etc.) Intensity: intervals with higher perceived intensity (B) Basic body awareness therapy: body scanning and stretch-release movements, postural stability, movement flow, and free breathing Both programs: 2 sessions/week, 1 h during which 5–8 participants trained at the same time Supervised by experienced physical therapists	Single consultation with advice on PA	CRF: VO2max, submaximal bicycle test	10 weeks	Improvements in MADRS score and cardiovascular fitness in the exercise group. Per-protocol analysis confirmed the effects of exercise and indicated that BBAT has an effect on self-rated depression
2014	Krogh et al. 2014 [84]	RCT MDD	41	38	Aerobic exercise intervention on stationary bikes Sessions: 3*x*/week, 45 min Intensity: 80% of their maximal heart rate Supervised: yes	Attention CG	CRF: *V*O2_max_, bicycle cardiopulmonary exercise test	12 weeks	Post-intervention the mean *V*O2_max_ increased with 3.90 ml/kg/min in the aerobic exercise group and 0.95 ml/kg/min in the control group The hippocampal volume, BDNF, VEGF, or IGF-1 did not differ between the two groups Positive association found post hoc between change in hippocampal volume and verbal memory and change in hippocampal volume and depressive symptoms
2014	Oertel-Knöchel et al., 2014 [37]	?? MDD and SZ	(A) 16 (B) 17	18	(A) CT combined with aerobic physical exercise: boxing, circuit training Intensity: 60–70% of individual HRmax (calculated from HRmax from ECG) Supervised by a trained physical exercise instructor (B) CT combined with relaxation training (no yoga or PMR, just breathing, “enjoy exercises”) Both 3*x*/week, 75 min: 30 min CT and 45 min training CT and relaxation conducted by an exercise instructor	Waiting list CG	Complete physical examination, ECG, blood investigation Validated questionnaires	4 weeks	Increase in cognitive performance in visual learning, working memory and speed of processing Increase in subjective quality of life between pre- and post-testing Significant reduction in depressive symptoms and state anxiety The effects in SZ patients compared with MDD patients were stronger for cognitive performance, whereas there were stronger effects in MDD patients than in SZ patients in individual psychopathology values
2012	Krogh et al. 2012 [93]	Outpatient RCT MDD	56	59	Aerobic exercise 3*x*/week: cycle ergometer Intensity: first 4 weeks: at least 65% of maximal capacity (*V*O2_max_), progressing to 70% and 80% during the second and third month, respectively Supervised by a physiotherapist	Stretching, low intensity	CRF: estimated VO2max, bicycle cardiopulmonary exercise test	12 weeks	After the intervention, the mean difference between groups was 20.78 points on the HAM-D17 At follow-up, higher *V*O2_max_ and visuospatial memory on Rey’s Complex Figure Test and lower blood glucose levels and waist circumference in aerobic exercise group compared with stretching exercise group
2010	Oeland et al. 2010 [94]	Controlled clinical study Panic disorder, generalized anxiety disorder, mild and moderate depression, mild and moderate recurrent depressive disorder	27	21	Group exercise: aerobic training (30 min) as circuit training Intensity: high intensity, at least 65–75% of maximum aerobic capacity AND Non-aerobic weightlifting with five basic exercises for muscles in legs, chest, abdomen, and lower and upper back: 8–10 repetitions with an intensity of 10 RM AND The instructor encouraged the participants to exercise once a week on their own initiative, at least 30 min; they were free to choose intensity and type of exercise 2*x*/week, 90 min Supervised: yes	Yes, but n.a	Aerobic capacity: submaximal bicycle ergometer test Muscle strength: Senior Fitness Test Questionnaires	20 weeks 12 weeks’ follow-up	Increase of physical activity and *V*O2_max_ in intervention group *V*O2_max_ increase was maintained after a 12-week follow-up period
2009	Krogh et al. 2009 [49]	RCT, outpatient Unipolar depression	(A) 55 (B) 55	55	(A) Strength group: circuit training with 6 machine exercises for large muscle groups Intensity: Initially 12 repetitions of 50% of RM 2 or 3 times per exercise. As the patients progressed, the numbers of repetitions were reduced to 10 and 8, and RM was increased to 75% (B) Aerobic group program: 10 different aerobic exercises for large muscle groups: cycling, running, stepping, etc Intensity: During the first 8 sessions, each exercise was done twice for 2 min at an intensity level of 70% of maximal heart rate and followed by a 2-min rest. This gradually increased to a level during the last 8 sessions at which each exercise was done for 3 min at an intensity level of 89%, with a 1-min rest Sessions: 2*x*/week, 1.5 h Supervised by a physiotherapist	Relaxation group	CRF: *V*O2_max_, bicycle ergometry; RM	4 months 6 months’ follow-up	Increase of strength measured by 1 RM in strength training group compared to relaxation group at month 4 Increase of *V*O2_max_ in aerobic group compared to relaxation group at month 4 No statistically significant effect on cognitive abilities
2009	Hoffman et al. 2008 [95]	RCT MDD	(A) 51 (B) 53 (C) 49	49	(A) Supervised aerobic exercise: 3*x*/week; individual training ranges equivalent to 70–85% HR reserve, calculated from the HRmax achieved during initial treadmill test (B) Home-based aerobic exercise: one initial training and 2 follow-up sessions with an exercise physiologist A and B: individual training ranges equivalent to 70–85% HR reserve, calculated from the HRmax achieved during initial treadmill test (C) Sertraline	Placebo pill	Aerobic capacity: *V*O2_peak_, graded treadmill exercise testing	16 weeks	Higher levels of *V*O2_peak_ and longer treadmill times in supervised exercise patients than in those who exercised at home No differences in neuropsychological tests between groups Better performance on tests of executive function but not on tests of verbal memory or verbal fluency/working memory in exercise group
2007	Legrand and Heuze 2007 [43]	Pilot study, randomized Depression	(A) 8 (B) 8	7	(A) High-frequency exercise: 3–5 sessions/week, within their THR on a motorized treadmill, a stationary bicycle, or a rowing ergometer (B) High-frequency exercise AND group-based intervention: 3–5 sessions/week AND group support: e.g., collective training sessions, asking participants to wear group T-shirts, encouraging participants to chat and to cheer each other on Supervision by first author of the study	Low-frequency exercise: 30 min/week of one aerobic exercise	Reaction to group intervention: participants’ scores of perceived cohesions Questionnaire sur l’Ambiance du Groupe	8 weeks	Lower depression scores in high-frequency aerobic exercise group than in CG at week 4 and 8 Alleviation in depressive symptoms was not found to be greater in those participants who received a group-based intervention
2002	Penninx et al. 2002 [112]	RCT, single blind Knee arthritis plus depression	(A) 112 low dep: 34 high (B) 115 low; 28 high	113 (36 high-dep)	(A) Resistance exercise: facility-based program, 3*x*/week, 1 h AND a 15-month home-based program. Repetitions of various upper and lower body exercises with dumbbells and cuff weights Supervised: yes (B) Aerobic exercise: indoor track; walking at an intensity equivalent to 50–70% of the HRR (determined from a screening exercise treadmill test). In months 4–6, the exercise leader visited participants four times and called them six times to offer assistance and support in the development of a walking exercise program in their home environment	Health education	Self-reported disability, 6-min walking speed, knee pain	3 months 15 months’ home-based follow-up	Significant decrease of depressive symptoms in aerobic exercise group compared to control group No such effect was observed for resistance exercise Reduction of depressive symptoms in both participants: with initially high and low depressive symptomatology Significant decrease of disability and pain and increase of walking speed in aerobic and resistance exercise group
1999	Blumenthal et al. 1999 [96]	RCT MDD	(A) 53 (B) 48 (C) 55		(A) Aerobic exercise session: 10 min warm up, 30 min continuous walking or jogging, 5 min cool down (B) Medication: antidepressants: Sertraline hydrochloride (C) Exercise + medications combined Sessions 3*x*/week Intensity: 70–85% of HRR calculated from HR_max_ Supervised: yes	n.a	Symptom-limited graded exercise treadmill test under continuous electrocardiographic recording	16 weeks	No statistical difference in groups on HAM-D or BDI scores Patients in the exercise and combination groups showed significant improvements in aerobic capacity, whereas patients in the medication group did not
1989	Martinsen et al. 1989 [45]	RCT Inpatient Depression	51	47	Aerobic exercise: Brisk walks and jogging Intensity: corresponding to approximately 70% of maximum aerobic capacity 3*x*/week, 1 h, 5–10 persons/group Supervision by an experienced instructor	Non-aerobic, low intensity, muscular strength training	CRF: *V*O2_max_, submaximal bicycle ergometer test	8 weeks	Significant increase of *V*O2_max_ in the aerobic group No change in the non-aerobic group Significant reduction of depression scores in both scores during the study Correlation between increase in physical fitness and reduction in depression scores was low

*BBAT* basic body awareness therapy, *BDI-II* Beck Depression Inventory-II, *BDNF* brain-derived neurotrophic factor, *CAT* continuous aerobic exercise training, *CG* control group, CRF cardiorespiratory fitness, *CT* cognitive training, *DASS-21* Depression Anxiety and Stress Scale-21, *ECG* electrocardiogram, *FI-Score* fitness Index Score, *HAM-D17* Hamilton Depression Scale-17, *HR*_*max*_ heart rate maximum, *HRR* heart rate reserve, *HRSD-17* Hamilton Rating Scale for Depression-17, *IG* Intervention Group, *IGF-1* insulin-like growth factor 1, *IPAQ* International Physical Activity Questionnaire, *IPAQ-SF* International Physical Activity Questionnaire–Short Form, *MADRS* Montgomery Asberg Depression Rating Scale, *MDD* Major depressive disorder, *MetS* Metabolic Syndrome, *n.a.* not applicable, *PA* Physical activity, *PMR* Progressive muscle relaxation, *RCT* randomized controlled trial, *RM* Repetition maximum, *SIT* sprint interval training, *THR* target heart rate, *UKK* 2 km Urho Kaleka Kekkonen 2-km Walk Test, *VAT* Ventilatory anaerobic threshold, *VEGF* vascular endothelial growth factor, *VO2*_*max*_ maximal oxygen uptake, *VO2*_*peak*_ peak oxygen uptake

In a randomized, controlled trial, 50 min’ add-on supervised aerobic exercise training 3 times a week for 4 months decreased symptoms of depression, anxiety, and stress compared with pharmacotherapy with antidepressants [41]. After an 8-week walking or running aerobic exercise program in local sports clubs, patients with MDD showed a large reduction in depressive symptoms compared with patients on a waiting list [42]. Moreover, an 8-week study found that high-frequency exercise was superior to low-frequency exercise with respect to depressive symptoms [43]. In an unsupervised study of physical activity in patients with MDD given access to fitness center resources, an increase in moderate-to-vigorous activity was associated with improvements in depressive symptoms [44]. In an 8-week study both aerobic and non-aerobic training methods had favorable effects on depression scores [45].

Different types of exercise have been studied in the last decade. A meta-analysis revealed small effects of aerobic exercise and yoga in outpatients with MDD, whereas the effects of Tai Chi were insufficient to enable conclusions to be drawn [46]. Additionally, aerobic exercise was superior to basic body awareness therapy with respect to depressive symptoms and cardiovascular fitness [47]. In patients with MDD randomized to 4 weeks’ sprint interval training or continuous aerobic exercise training, improvements in CRF were observed in both groups and were associated with improved depressive symptoms, emotional wellbeing, and sleep [48]. In contrast, another study found no improvements in depression score in the Hamilton Rating Scale for Depression after a 4-month strength and aerobic exercise training in patients with MDD [49]. Using mendelian randomization methods on genomic and phenotypic data from the UK biobank, beneficial effects of exercise were detected in depression but not in SZ [50, 51] (Table 1).

### Effects of physical activity on symptoms of BD

No interventional studies have examined the effects of exercise in patients with BD. However, in a study examining the effects of *N*-acetylcysteine treatment, physical activity was not related to improvements in depressive symptoms, although those participants who engaged in higher levels of physical activity had greater improvements in social and occupational functioning [52].

### Aerobic exercise training improves cognition and symptoms in patients with SZ

Several studies have demonstrated beneficial effects of physical exercise on symptoms of SZ (Table 2). For example, a well-cited meta-analysis showed that in patients with SZ aerobic exercise improves negative, positive, and depressive symptoms and global functioning, as measured by the Global Assessment of Functioning (GAF) score [53]. In addition, another meta-analysis focusing on cognition demonstrated improved global cognition, working memory, social cognition, and attention after aerobic exercise in patients with SZ [54]. A recent meta-analysis of randomized controlled trials found that aerobic exercise had small beneficial effects on negative symptoms in patients with SZ [55]. Across aerobic exercise studies, symptom improvement was seen in interventions consisting of 90 min of moderate exercise per week [56]. This finding is in line with our own work, which showed that 3 × 30 min of aerobic exercise per week alleviated negative symptoms and significantly improved global functioning and short-term memory in patients with SZ [57, 58]. Moreover, we found preliminary evidence that the improvements in level of functioning might be sustained even after exercise cessation [59].

**Table 2:** Aerobic exercise studies in schizophrenia, including methodology, cardiorespiratory fitness measurements, and clinical outcome

Year	Publication	Study design and diagnosis	Sample size, *n*		Intervention	Control	Measurement of CRF and PA	Intervention duration /follow-up	Outcome
			IG	CG					
2021	Kimhy et al. 2021 [105]	Single-blind RCT SZ	16	17	Aerobic exercise program with 2 active-play video games (Xbox 360 Kinect) and traditional aerobic exercise equipment (treadmill, bike) Sessions: 3*x*/week, 1 h Moderate intensity: activities that expend 3.0–5.9 times the energy expended at rest; 60% of HR_max_ in week 1, 65% in week 2, 70% in week 3, and 75% in weeks 4–12 Supervision by a BSc in Science of Therapeutic Recreation	UC	CRF: *V*O2_peak_, cycle ergometer	12 weeks	↑ In *V*O2_max_ by 18.0% vs. 0.5% Improvements in *V*O2_max_ significantly predicted enhancement in SF as indexed by self-, informant-, and clinician-reported measures, predicting 47%, 33%, and 25% of the variance, respectively Significant improvement in SF (23.0% vs. − 4.2%)
2020	Andersen et al. 2020 [99]	RCT SZ	21	26	HIIT Walking/running on a treadmill, Sessions: 2*x*/week, 45 min Intensity: 4 × 4 min 85–95% of HR_max_, active breaks consisting of 3 min of ~ 70% of HR_max_ Supervision by mental health care providers with or without PA competence (half of the participants, respectively)	PC gaming skills (Nintendo Wii sports console), supervised	CRF: *V*O2_max_ (treadmill, maximum exercise session, mod. Balke protocol) PA: Actigraph GT3X + accelerometer	12 weeks	No significant within-group differences in CRF ↑ Workload in 61% of HIIT ↑ In *V*O2_max_ when adding PA competence of the mental health care providers No significant effect on PA level or body composition
2020	Dubreucq et al. 2020 [113]	Quasi experimental trial SZ	57	30	Exercise-enriched integrated social cognitive remediation intervention Sessions: 1*x*/week, 2 h Intensity: not defined Supervised by two facilitators	Active CG practicing Touch Rugby 12 × 2-h sessions Supervised by specialized sport scientists		12 weeks 6 months’ follow-up	Moderate to large improvements in social function, symptom severity, verbal abstraction, aggression bias, and self-stigma that were specific to the IG and were not observed in participants playing only Touch Rugby. Effects were persistent over time and even larger between post-treatment and follow-up
2020	Korman et al. 2020 [102]	Single arm, prospective feasibility study SMI; 92% SZ	42	No CG	Mixed aerobic and resistance training: circuit training, combined with a dietary intervention (six individual and group sessions) Sessions: 3*x*/week, 1 h Intensity: Moderate from week 3. (RPE at 2–3 for the first 2 weeks, then increased to a minimum RPE of 4/10 by week 3 and further increased as per individual participant's capacity) Supervised by exercise physiology students	No CG	Functional exercise capacity: 6 MWT PA: questionnaire SIMPAQ Motivation towards exercise: BREQ	10 weeks	Significant improvements in functional exercise capacity, volume of exercise, general psychiatric symptoms, and negative psychotic symptoms No change in anthropometric and metabolic blood markers
2020	Massa et al. 2020 [101]	RCT Outpatient SZ	21	17	Aerobic exercise on a stationary bicycle, groups of about 5 participants Sessions: 3*x*/week, initially 20 min to 45 min (increasing 5 min each week) Intensity began at low levels (50% of maximal HRR) to 80% of HR_max_ (increased by 5% every week) Supervised by at least one qualified instructor	Stretching and balance training for same amount of time, groups of about 5 participants	400 m walk test → Estimate *V*O2_max_	12 weeks 8 weeks’ follow-up	Subjects in both groups were slower at the 400 m walk in week 12 compared with baseline, but the IG had significantly less slowing than the CG Between week 12 and week 20, the aerobic exercise group had a significantly greater change score on the Composite and Visual Learning Domain of the MATRICS Consensus Cognitive Battery
2019	Brobakken et al. 2019 [100]	RCT Feasibility study SZ	25	23	Aerobic interval training in groups, walking/running on a treadmill Sessions: 2*x*/week, 35 min Intensity: 4 × 4 min 85–95% of HRpeak, active pauses consisting of 3 min of ~ 70% of HR_peak_ Supervised by experienced healthcare professionals	Two aerobic interval training sessions and encouragement to exercise on their own	CRF: *V*O_2peak_	12 weeks	↑*V*O2_peak_ by 10%, no change in the CG No intergroup difference in weight, body mass index (BMI), waist circumference, blood pressure, lipids, or glucose at posttest ↑Weight and BMI in the CG, no change in the IG
2019	Hallgren et al. 2019 [60]	Single-arm feasibility study First-episode psychosis; majority SZ	91	No CG	Circuit training: high-volume resistance exercises, aerobic training, and stretching Sessions: at least 3*x*/week, 1 h Supervised by exercise science graduates and exercise physiologist	No CG		12 weeks	Significant post-intervention improvements for processing speed, visual learning, and visual attention; all with moderate effect sizes
2019	Larsen et al. 2019 [114]	RCT, Feasibility study First-episode psychosis, SZ, Schizotypal and delusional disorder, and other non-organic psychotic disorders	13	12	Cross-fit-oriented training Session: 3*x*/week, 1 h Moderate to high intensity Supervised by two instructors (undergraduate students)	Waiting list CG		8 weeks	Three main themes and ten subthemes emerged during the analysis: (1) motivation and expectations for enrollment (subthemes: routines and structure, social obligation, goal setting and self-worth); (2) new demands and opportunities (subthemes: practicalities of the training, an understanding exercise setting, and alone and together); and (3) looking ahead—reflections on impact (subthemes: restored sleep and circadian rhythm, energy and sense of achievement, changed everyday life, and hope of finding a new path)
2019	Shimada et al. 2019 [62]	Pilot RCT SZ	(A) 16	15	(A) Aerobic exercise: individual and group programs Sessions: 2*x*/week, 1 h Intensity: individually calibrated at 60–80% of aerobic capacity. Patients were required to participate in a minimum of 75% of each session Supervised by occupational therapists	UC	n.a	12 weeks	IG and CG patients showed significant improvements in cognition, intrinsic motivation, psychiatric symptoms, and interpersonal relations
2017	Bhatia et al. 2017 [115]	Single-blind RCT SZ	A) 104 (B) 90	92	(A) Yoga training (postures and breathing) (B) Physical exercise training: brisk walks, jogging, and directed aerobic exercises Sessions: 5*x*/week, 1 h Supervised by qualified instructors	UC	n.a	21 days 6 months’ follow-up (provided with a yoga training booklet, compliance charts)	Speed index of attention domain in group (A) showed greater improvement than group (B) at 6 month follow-up In group (B), accuracy index of attention domain showed greater improvement than UC alone at 6-month follow-up For several other cognitive domains, significant improvements were observed with (A) or (B) compared with UC alone
2017	Cheng et al. 2017 [107]	RCT SZ	26	28	Aerobic dance program Sessions: 2*x*/week, 60 min Intensity: 60–79% of predicted HR_max_ Supervised by professional instructor	UC	Muscular endurance (1-min flexed leg sit-up), flexibility (sit-and-reach test), cardiorespiratory endurance (3-min step test; HR)	8 weeks 4 weeks’ follow-up	Significant between-group differences at posttest and in the follow-up for all of the health-related fitness outcomes with the exception of muscular endurance
2017	Curcic et al. 2017 [63]	RCT SZ	40	40	Individual training: Walking/running 2–4 km outside Sessions: 4*x*/week, 45 min Intensity: 65–75% HR_max_ Supervised by a fitness trainer	UC	CRF: *V*O_2max_	12 weeks	After 12 weeks, patients in IG showed a significant increase of *V*O2_max_ and significantly higher level of *V*O2_max_ compared to the CG Significant differences on PANSS general psychopathology subscale and on PANSS total score. The pharmacotherapy and exercise had influence on PANSS general psychopathology and PANSS total score
2016	Duncan et al. 2016 [116]	Randomized cross-over study SZ	28		Bout of exercise: walking on the treadmill Intensity: moderate (64–76% of the calculated HR_max_) 1 × 10 min	Passive sitting for 14 min	Mean HR, percent maximum HR, Borg RPE	10-min post-tests after 14 and 24 min	Significant differences between pleasure at baseline, both immediately after task and 10 min after task. No other main effects or interactions
2016	Ho et al. 2016 [68]	RCT Chronic SZ	(A) 51 (B) 51	49	(A) Tai Chi: 22 simple movements (B) Moderate aerobic exercise routine to achieve 50–60% of maximal oxygen consumption Both groups: 1*x*/week, 60 min AND 2*x*/week, 45 min Supervised by mental health professionals	Wait list control UC; they were offered the Tai chi or exercise class on a voluntary basis after the 3-month post-intervention follow-up assessment	Heart rate	12 weeks 3 months’ follow-up	Compared with CG, the Tai-chi group showed significant decreases in motor deficits and increases in backward digit span and mean cortisol, while the exercise group displayed significant decreases in motor deficits, negative and depression symptoms and increases in forward digit span, daily living function, and mean cortisol No significantly different therapeutic effects of the two interventions, except for fewer symptom manifestations in the exercise group
2016	Kang et al. 2016 [67]	RCT Chronic SZ	118	126	Community-based integrated intervention = Tai Chi Intervention AND Social Skills Training Sessions 2*x*/month, 120 min: 45 min social skill training, 45 min Tai Chi, 30 min break Supervised by three full-time psychiatrists and one assistant Both groups received medication maintenance treatment to prevent relapse	Medical treatment alone		12 months	Compared with the medical treatment alone group, the community-based integrated intervention group had lower scores on PANSS and negative symptoms, a lower risk for aggressive behavior, and a greater improvement in adherence to medication after 1 year of intervention
2016	Keller-Varady et al. 2016 [29]	Controlled interventional study SZ	(A) 22 (B) 21	22 healthy controls	(A) Endurance group: dynamic aerobic endurance training on bicycle ergometers Sessions: 3*x*/week, 30 min Intensity: set according to the individual results of a baseline assessment of endurance capacity, equivalent to blood lactate concentrations of 2 mmol/l Supervised by sport scientist (B) Table soccer group: table soccer Sessions: 3*x*/week, 30 min Supervision: n.a All participants continued with their usual medication	Healthy control in endurance group	Endurance capacity by ergometer stress test Standardized questionnaire, specially developed for measuring PA	12 weeks 3 and 6 months’ follow-up	Improvements of endurance capacity in (A), but not in (B) Patients and healthy controls showed comparable adaptations to endurance training, as assessed by physical working capacity and maximal achieved power Differences in changes of performance at a lactate concentration of 3 mmol/l
2016	Malchow et al. 2016 [88]	RCT SZ	2021 healthy controls	19	Endurance training on bicycle ergometers Sessions: 3*x*/week, 30 min Intensity: individually defined intensity, gradually increased according to blood lactate concentrations of approximately 2 mmol/l, HR, Borg Scale Supervised by sports scientists From week 6, the computer-assisted training program COGPACK was added as an intervention in each group to train cognitive performance	Table soccer in groups (2–4 players)	Endurance capacity (PWC130) by maximal exercise stress test PA was monitored throughout the study, not defined	6 weeks 3 months	No significant increases in the volumes of the hippocampus or hippocampal substructures in SZ patients or healthy controls Increased volume of the left superior, middle, and inferior anterior temporal gyri compared with baseline in SZ patients after the endurance training, whereas patients playing table soccer showed increased volumes in the motor and anterior cingulate cortices. After the additional training-free period, the differences were no longer present Improvements of endurance capacity in exercising patients and healthy controls No change in psychopathological symptoms
2016	Su et al. 2016 [61]	3-month follow-up study, single blind, randomized SZ	30	27	Aerobic exercise: individually tailored for each participant because exercise prescriptions were based on each individual's age-adjusted HR_max_ Sessions: at least 3*x*/week, 40 min One-on-one supervision throughout the sessions	Stretching and toning control group, individually conducted, own pace Same social interactions as those in aerobic exercise group	Estimated *V*O2_max_	12 weeks 3 months’ follow-up	No significant difference between the two groups in any cognitive outcome measured at follow-up; improvement over time was noted in certain cognitive domains in the IG No significant between-group differences in aerobic fitness at posttest and follow-up Fitness level was not related to changes in cognitive performance
2016	Yoon et al. 2016 [108]	Single-arm pilot study SZor schizoaffective disorder	24	No CG	Exercise intervention: group-based outdoor cycling 1*x*/week, at least 40 min Individual performance (average speed, distance, and duration) and heart rate, were monitored by individual supervising staff during every session.) Supervised by two professional cyclists and other staff: medical doctors, nurses, and social workers	No CG	Cardiorespiratory function test (step test): 3-min YMCA step test	3 months 6 months’ follow-up	Significant increase in participant’s self-esteem, positive relationship, global function, and quality of life CRF significantly improved after 3 months At the 9-month follow-up, 6 months after program completion, only in interpersonal relationship change the improved effects were maintained
2015	Kaltsatou et al. 2015 [69]	RCT, outpatient SZ	16	15	Greek traditional dancing program Sessions: 3*x*/week, 60 min Intensity: 60–70% of individual HR_max_ (220-age) Supervised by a PA instructor	Sedentary CG, Patients were asked to refrain from any other form of organized PA during study period	6 MWT Sit-to-stand test, 10 times Lower limb strength testing Hand-grip strength	8 months	↑ Walking distance in the 6 MWT, sit-to-stand test, Berg Balance Scale score, lower limbs maximal isometric force, Positive and Negative Syndrome Scale total score, Global Assessment of Functioning scale total score, and Quality of Life total score
2015	Kimhy et al. 2015 [117]	Single-blind RCT, inpatient SZ	16	17	Aerobic exercise program utilizing 2 active-play video games (Xbox 360 Kinect) and traditional aerobic exercise equipment (treadmill, bike) Sessions: 3*x*/week, 1 h Intensity: moderate intensity, minimal aerobic exercise intensity was set to 60% of HR_max_ in week 1, 65% in week 2, 70% in week 3, and 75% in weeks 4–12 Supervised by a BSc in Science of Therapeutic Recreation	UC	CRF: *V*O2_peak_	12 weeks	↑ *V*O2_peak_ by 18.0% in the IG vs a − 0.5% decline in the CG Improvement of neurocognition by 15.1% vs − 2.0% CRF and increases in BDNF predicted 25.4% and 14.6% of the neurocognitive improvement variance, respectively
2015	Loh et al. 2015 [118]	RCT, inpatient SZ	52	52	Structured, organized walking intervention, 3*x*/week, supervised 3*x*/week, the first month: 20-min walking exercise, second month: 30-min walking exercise, third month: 40-min walking HR was monitored before and after the exercise to prevent overexertion Supervised by ward staff nurses and assistant medical officers	Treatment as usual	IPAQ-M	12 weeks 3 months’ follow-up	At 3-month follow-up, significant within-group differences in QOL (SF-36), psychiatric symptoms (PANSS), and personal and social performance (PSP) Increase in the median SF-36 scores, with increases shown in physical functioning, physical role limitations, social functioning Reduction of median PANSS in positive and negative symptom, and general psychopathology scales Increase in the median PSP score Between-group differences at post-intervention (favoring intervention) were significant for PANSS positive and SF-36
2015	Malchow et al. 2015 [58]	RCT SZ	2223 healthy controls	21	Endurance training on bicycle ergometers Sessions: 3*x*/week, 30 min Intensity: individually defined intensity, gradually increased according to blood lactate concentrations of approximately 2 mmol/l, HR, Borg Scale Supervised by sports scientists From week 6, the computer-assisted training program COGPACK was added as an intervention in each group to train cognitive performance	Table soccer in groups (2–4 players)	Endurance capacity (PWC130) by a maximal exercise test on a bicycle ergometer	6 weeks 3 months 6 months’ follow-up	After 3 months, improvement in GAF and SAS-II social/leisure activities and household functioning adaptation in the endurance training augmented with cognitive remediation, but not in the table soccer augmented with cognitive remediation group Significant improvements in the severity of negative symptoms and performance in the VLMT and WCST in the endurance training augmented with cognitive remediation group from week 6 to the end of the 3-month training period
2015	Masa-Font et al. 2015 [111]	RCT Severe mental illness; 67% SZ	169	163	Educational program AND PA program based on different stages → 24 sessions 2*x*/week over 3 months: The first 8 sessions (40 min) consisted of making first contact with PA. The other 16 sessions (60 min) aimed to increase the number of daily steps taken to reach 10,000 steps per day on routes adapted to the physical condition of the participants AND Dietary intervention → 16 sessions 2*x*/week, 20 min to provide basic knowledge on healthy dietary habits Supervised by professionals	No intervention, usual program of regular check-ups with their reference psychiatrist All the participants in both the IG and CG kept up their usual visits with their reference mental health professional and continued the usual treatment for their disease	PA: IPAQ, METs expended per week	3 months 12 months’ follow-up	↑ Average weekly walking METs in the IG BMI decreased significantly more in the CG No significant differences in the waist circumference
2015	Silva et al. 2015 [70]	RCT “blind” SZ	(A) 12 (B) 9	13	(A) Resistance: progressive resistance training Intensity: from 40% 1RM in week 1 up to 85% 1RM in week 20 (B) Concurrent exercise: endurance training and strength resistance training Intensity: from 40% *V*O2_max_ in week 1 up to 75% *V*O2_max_ in week 20 Sessions: 2*x*/week, 60 min Supervised by professional physical educators	Control: equipment load was kept at a minimum, treadmill speed remained at 4 km/h	CRF: *V*O2_max_	20 weeks	A significant time-by-group interaction was found for (A) and (B) on the Positive and Negative Syndrome Scale total score for disease symptoms, positive symptoms, and on the arm extension one-repetition maximum test Improvements in (A) on negative symptoms, on the role-physical domain of the Short Form-36 Health Survey, and on the chest press 1RM test
2015	Svatkova et al. 2015 [90]	RCT SZ	(A) 16 (B) 17	(A) 24 (B) 24 healthy controls	(A) Aerobic and anerobic exercise. Aerobic exercise: bicycle ergometer, rowing machine, cross trainer, treadmill and muscle strength exercises (6 exercises per week, 3 times). Anaerobic exercise: working with weights 2*x*/week, 1 h: 40 min aerobic training, 20 min anaerobic training Intensity: n.a Supervision: n.a (B) No exercise = life-as-usual	(A) exercise (B) no exercise = life- as-usual Healthy controls	CRF: *V*O2_peak_ Structured questionnaire to assess the level of daily life physical activities at baseline Brain scans	6 months	Irrespective of diagnosis, regular physical exercise of an overlearned skill, such as bicycling, significantly increases the integrity, especially of motor functioning-related white matter fiber tracts, whereas life-as-usual leads to a decrease in fiber integrity Significant differences in the exercise and non-exercise group from the first to the second measurement in Wpeak and *V*O2_peak_
2014	Oertel-Knöchel et al. 2014 [37]	MDD (*n* = 22) and SZ (*n* = 29)	(A) 16 (B) 17	18	(A) Cognitive training combined with aerobic physical exercise: boxing, circuit training Intensity: 60–70% of individual HRmax (calculated from HR_max_ from ECG) Supervised by a trained physical exercise instructor (B) CT combined with relaxation training (no yoga or PMR, just breathing, “enjoy exercises”) CT and relaxation conducted by instructors Both sessions: 3*x*/week, 75 min (30 min CT and 45 min training)	Waiting list CG		4 weeks	Increase in cognitive performance in the domains visual learning, working memory and speed of processing; decrease in state anxiety; and increase in subjective quality of life in the total group of patients The effects in SZ patients compared with MDD patients were stronger for cognitive performance, whereas there were stronger effects in MDD patients compared with SZ patients in individual psychopathology values. MDD Reductions in depressive symptoms and state anxiety values in patients Reduction of negative symptoms severity in SZ patients
2014	Vancampfort et al. 2014 [119]	Pilot Study cross-sectional SZ	88		No intervention		Spirometry: 2 spirometry attempts while seated, conducted by trained technicians CRF: 6-min walk test PA: IPAQ	Screening: 6 months	Patients with MetS had a reduced predicted forced expiratory volume for 1 s and predicted forced vital capacity Significantly more patients with MetS were diagnosed with restrictive lung dysfunction SZ patients with restrictive lung dysfunction had a significantly larger waist circumference, were less physically active and walked less on the 6 MWT than patients without
2013	Scheewe et al. 2013 [64]	RCT SZ	31	32	Exercise therapy: Muscle strength exercises (six exercises/week, 3 × 10 to 15 RM for biceps, triceps, abdominal, quadriceps, pectoral, and deltoid muscles). 2*x*/week, 1 h Intensity: increased stepwise: week 1–3, 45%; week 4–12, 65%; week 13–26, 75% of HR reserve based on baseline CPET data Supervised by a psychomotor therapist	Occupational therapy creative and recreational activities	CRF: *V*O2_peak_ and *W*_peak_	6 months	↑ *W*_peak_ in IG compared with CG Exercise therapy reduced symptoms of SZ, depression, need of care, and increased *V*O2_peak_ in the IG compared with CG No effect for MetS factors
2013	Scheewe et al. 2013 [85]	RCT SZ	(A) 18 (B) 14	(A) 25 (B) 27	(A) Exercise therapy intervention: upright and recumbent bicycle ergometer, rowing machine, cross trainer, and treadmill AND muscle strength exercises (for biceps, triceps, abdominal, quadriceps, pectoral, deltoid muscles) 1 h of exercise consisting of both cardiovascular exercises (40 min) and muscle strength exercises (20 min) twice weekly Supervised by a psychomotor therapist specialized in psychiatry (B) Occupational therapy by an occupational therapist 1 h/twice weekly: Occupational therapy comprised creative and recreational activities, no physical activity	(A) Exercise therapy (B) Life as usual More details n.a	CRF: *V*O2_peak_	6 months	Significantly smaller baseline cerebral (gray) matter, and larger third ventricle volumes, and thinner cortex in most areas of the brain in patients versus controls No changes in global brain and hippocampal volume or cortical thickness CRF improvement was related to increased cerebral matter volume and lateral and third ventricle volume decrease in patients and to thickening in the left hemisphere in large areas of the frontal, temporal and cingulate cortex irrespective of diagnosis 1–2 h of exercise therapy did not elicit significant brain volume changes in patients or controls CRF improvement attenuated brain volume changes
2012	Scheewe et al. 2012 [106]	RCT SZ	(A) Exercise therapy 31// Healthy controls 27 (B) Occupational therapy 32	28	Exercise therapy: (A) Muscle strength exercises (six exercises weekly; three times 10–15 RM for biceps, triceps, abdominal, quadriceps, pectoral, and deltoid muscles) Sessions: 2*x*/week, 1 h Intensity was increased stepwise (week 1–3, 45%; week 4–12, 65%; week 13–26, 75% of HRR based on baseline CPET data Supervised by a psychomotor therapist (B) Patients not randomized to exercise therapy were offered occupational therapy 2*x*/week, 1 h: creative and recreational activities	Life as usual, not allowed to incorporate moderate physical activity more than 1 h weekly	CRF: *V*O2_peak_ and *W*_peak_	6 months	Patients had higher resting HR and lower peak HR, peak systolic blood pressure, relative VO2peak, Wpeak, RER, minute ventilation and HR recovery than controls In patients, exercise therapy increased relative *V*O2_peak_ compared with decreased relative *V*O2_peak_ after occupational therapy In controls, relative *V*O2_peak_ increased after exercise therapy and to a lesser extent after life-as-usual Exercise therapy increased *W*_peak_ in patients and controls compared with decreased *W*_peak_ in nonexercising patients and controls
2012	Takahashi et al. 2012 [87]	?? SZ	13	10	Program including exercise, nutrition education and medication counseling Exercise module: aerobic exercise (walking and jogging), muscle-stretching exercise and sports exercise (basketball). The exercise module was 30–60 min long, and was delivered twice a day (a total of 60–120 min per day) from Monday to Saturday For each participant, the exercise intensity level was set at 11–13 (fairly light to somewhat hard) on the Borg scale Supervised by a group of professionals from diverse disciplines (physical therapists, psychiatric nurses, psychiatrists, nutritionists, and pharmacists) All patients received antipsychotics, and their medications remained unchanged during this study	Attended the day-hospital unit but not the program		3 months	Body mass index and general psychopathology scale of PANSS were significantly reduced in the program group but not in the control group after a 3-month interval Compared with baseline, activation of the body-selective extrastriate body area [23] in the posterior temporal-occipital cortex during observation of sports-related actions was increased in the program group. In this group, increase in EBA activation was associated with improvement in the general psychopathology scale of PANSS Sports participation had a positive effect not only on weight gain but also on psychiatric symptoms in schizophrenia
2011	Heggelund et al. 2011 [104]	RCT SZ	12	7	HIIT Sessions: 3 days/week, 36 min: 4 × 4 min Intensity: 4 min 85–95%, 3 min 70% HR_peak_ Supervised by an exercise physiologist	Played PC (Tetris) games; 3 days/week, 36 min Supervised by the same exercise physiologist	CRF: *V*O2_peak_	8 weeks	The HIIT group improved VO2peak by 12% compared with the CG group Net mechanical efficiency of walking improved 12% in the HIIT group compared with the CG group No significant changes in PANSS, CDSS or SF-36 in either group
2011	Methapatara and Srisurapanont 2011 [120]	RCT SZ	32	32	Pedometer walking AND motivational interviewing program → 5 × 1-h sessions: First session: individual motivational interviewing with a focus on obesity/overweight and motivation for adequate daily walking Second session: group education on nutrition, exercise, warming up, cooling down, and implementation of pedometer Third session: specific, measurable, acceptable, realistic, and timed criteria were used to set an individual goal, the first goal of daily walking was set at a minimum of 3000 steps per day Forth session: group practicing of pedometer walking under supervision Fifth session: therapist gave feedback on the patients’ practice, informed about self-regulation principles to cope with lapses and relapse	Control patients received the usual care only and no pedometer		12 weeks	Bodyweight of intervention group decreased more than that of the control group at week 4, 8, and 12 BMI at week 12 was significantly different between groups The decreases of waist circumference were significantly more in the intervention group for all three time-points of assessment
2011	Vancampfort et al. 2011 [65]	Pilot study SZ	(A) 40 (B) 40	40	(A) One single 30-min yoga session, trained by a physiotherapist (B) One single 20-min aerobic exercise session, performed on an electronically braked ergometer; consisted of cycling for 20 min at self-selected intensity with heart rate feedback. A physiotherapist was present during exercise	No exercise, participants sat quietly in a room for 20 min and were allowed to read. A physiotherapist was also present	n.a	One single event	After single sessions of yoga and aerobic exercise, individuals with SZ or schizoaffective disorder showed significantly decreased state anxiety, decreased psychological stress, and increased subjective wellbeing compared to a no-exercise control condition. The magnitude of the changes did not differ significantly between yoga and aerobic exercise
2010	Pajonk et al. 2010 [57]	RCT, day-hospital/outpatient SZ	88 healthy control	8	Aerobic exercise training (cycling) Sessions: 3*x*/week, 30 min Intensity: heart rate (± 10 beats/min) corresponding to a blood lactate concentration of about 1.5–2 mmol/l (14–18 mg/dl) derived from the results of the pretest Supervised by one of the investigators	Played table football The comparison group of patients played tabletop football for 30 min, 3 times per week, in a setting with comparable levels of stimulation to those provided for aerobic exercise. Tabletop football enhances coordination and concentration but does not improve aerobic fitness	CRF: VO2max	3 months	After exercise training, relative hippocampal volume increased significantly in patients (12%) and healthy individuals (16%), with no change in the non-exercise group of patients Changes in hippocampal volume in the exercise group were correlated with improvements in aerobic fitness measured by change in maximum oxygen consumption In the SZ exercise group, change in hippocampal volume was associated with a 35% increase in the *N*-acetylaspartate to creatine ratio in the hippocampus Improvement in test scores for short-term memory in the combined exercise and non-exercise SZ group was correlated with change in hippocampal volume

*BDNF* brain-derived neurotrophic factor, *BMI* body mass index, *BREQ* Behavioral Regulation In Exercise Questionnaire, *CDSS* Calgary Depression Scale for Schizophrenia, *CG* control group, *CPET* cardiopulmonary exercise testing, *CRF* cardiorespiratory fitness, *CT* cognitive training, *EBA* extrastriate body area, *GAF* Global Assessment of Functioning, HIIT high-intensity interval training, *HR* heart rate, *HR*_*max*_ heart rate maximum, *HR*_*peak*_ peak hear rate, *HRR* Heart rate reserve, *IG* Intervention group, *IPAQ* International Physical Activity Questionnaire, *IPAQ-M* International Physical Activity Questionnaire, Malay version, *MATRICS* Measurement and Treatment Research to Improve Cognition in Schizophrenia, *MDD* Major depressive disorder, MET metabolic equivalent, *MetS* Metabolic Syndrom, *MWT* Minute walk test, *n.a.* not applicable, *PA* Physical activity, *PANSS* Positive and Negative Syndrome Scale, *PMR* progressive muscle relaxation, *PSP* personal and social performance, *PWC130* Physical Working Capacity 130, *QoL* Quality of Life, *RCT* Randomized controlled trial, *RER* respiratory exchange ratio, *RM* Repetition maximum, *RPE* Rating of perceived exertion, *SAS II* Social Adjustment Scale-II, *SF-36* Short-Form-36, *SIMPAQ* Simple Physical Activity Questionnaire, *SZ* schizophrenia, *UC* usual care, *VLMT* verbal learning memory test, *VLMT* verbal learning memory test, *VO2*_*max*_ maximal oxygen uptake, *VO2*_*peak*_ Peak oxygen uptake, *WCST* Wisconsin Card Sorting Test, *W*_*peak*_ Watt peak, *WSCT* Wisconsin Card Sorting Test, *YT* Yoga Training

Effects of aerobic exercise on cognition have been observed also in patients with first-episode SZ. After a 12-week supervised circuit-training program, improvement was seen in processing speed, visual learning, and visual attention domains [60]. In 75 patients with SZ randomized to 12 weeks of either moderate-intensity treadmill exercise or stretching and toning exercise, aerobic exercise improved processing speed and attention [61]. However, in a pilot randomized controlled trial in a small sample, group aerobic exercise over 12 weeks showed similar improvements in cognition and symptoms as treatment as usual [62]. After patients with SZ performed 12 weeks of treadmill training, their general and psychopathology and total score on the Positive and Negative Symptom Scale (PANSS) and aerobic capacity improved [63]. In a 6-month randomized study comparing aerobic exercise with occupational therapy in patients with SZ, exercise reduced symptoms, depression, and need of care and increased cardiovascular fitness [64]. In contrast, after a single session of aerobic exercise and yoga, patients from both groups showed only decreased anxiety and psychological stress and increased subjective wellbeing [65]. In meta-analyses of meditation-based mind–body interventions, small effect sizes have been observed for yoga in SZ [66]. Besides yoga, other exercise interventions such as Tai Chi have been applied in SZ patients and led to improvements in PANSS score, negative symptoms, and aggressive behavior [67]. A study that compared a 12-week Tai Chi program with aerobic exercise showed improved negative and depression symptoms [68]. In a randomized 8-month study of a Greek traditional dancing program, the dancing group showed improved positive and negative symptoms, GAF score, and quality of life compared with a sedentary group [69]. Finally, resistance training was studied in patients with SZ and improved negative symptoms [70] and level of functioning assessed with the GAF [71] (Table 2).

### Neuroplasticity effects of aerobic exercise

Animal models and basic research in humans clearly show that aerobic exercise has favorable neurobiological effects. These effects may involve epigenetic alterations, synaptic plasticity, differentiation of glial cells and neurogenesis, the hypothalamus–pituitary–adrenal axis, growth factors, immune-related mechanisms, neurotransmitters, and the endocannabinoid system [72]. In 2103 adults from the general population, CRF, measured as peak oxygen uptake (*V*O2_peak_), was related to higher gray matter volume and showed a strong association with gray matter volume of the left middle temporal gyrus, right hippocampus, left orbitofrontal cortex, and bilateral cingulate cortex [73]. A meta-analysis of hippocampal volume in 737 voluntary participants revealed significant positive effects of aerobic exercise on left hippocampal volume but not on total hippocampus volume [74]. These results may be relevant for MDD, BD, and SZ because these brain disorders have been repeatedly shown to involve structural and functional alterations in the hippocampal formation [75, 76]. Moreover, a 7-Tesla magnetic resonance imaging study in older adults found a prominent volume increase in the left cornu ammonis (CA) subregions of the hippocampus and a trend for a volume increase in the left CA4/dentate gyrus after physical activity [77].

Deficits in both episodic and working memory are related to hippocampal abnormalities and are hallmarks of an unfavorable outcome in MDD [78] and SZ [79]. Our first study to investigate the effects of aerobic endurance training in a small sample of patients with multi-episode SZ showed a significant 10% increase in hippocampal volume after 3 months [57]. In our subsequent study in 20 patients, which combined 3 months of aerobic endurance training with cognitive remediation, we found no changes in hippocampal volume in the exercise group [58], but we did find a significant correlation between exercise-related volume increases in the CA4/dentate gyrus subregion of the hippocampus and the SZ polygenic risk score (SZ-PRS, [80]). Using cell-specific PRS, we found that this volume effect in CA4 was also caused by oligodendrocyte precursor cell-related pathways [81], which is also in line with our post-mortem finding of reduced oligodendrocyte number in the CA4 subregion [82]. In SZ and MDD it has been hypothesized that metabolic coupling may link oligodendrocyte to interneuron pathology [83]. Other studies found no changes in total hippocampal volume after aerobic exercise in MDD [84] or SZ [85]. However, after a 12-week aerobic exercise training, hippocampal volume in the CA1 subregion increased in SZ patients, whereas hippocampal vascular volume was unchanged, indicating no effect of aerobic exercise on blood vessels [86]. Additionally, a study that compared aerobic exercise training with table soccer in patients with SZ and healthy controls showed an increased volume of the right entorhinal cortex compared with baseline after 6 weeks’ training [87] and of the left superior, middle, and inferior anterior temporal gyri after 3 months’ training; but patients with SZ who played table soccer showed increased volumes in the motor and anterior cingulate cortices [88]. After 6 weeks’ aerobic exercise training, a magnetic resonance spectroscopy study in patients with SZ found increased *N*-acetyl-aspartate/total creatine levels in the left dorsolateral prefrontal cortex in both the aerobic exercise and table soccer groups [89], indicating improved neuronal viability. Additionally, a 6-month aerobic exercise program improved the integrity of motor function-related white matter fiber tracts compared with a life-as-usual condition [90].

Taken together, these results indicate that in SZ exercise has neuroplastic effects in brain regions that are affected by the disease itself. The effects of aerobic exercise on brain volume changes and underlying mechanisms warrant further study, not only in patients with SZ but also in those with MDD and BD.

### Improvements of CRF in patients with severe mental illness

CRF is an important marker of cardiovascular health and should be comprehensively assessed in both clinical studies and clinical practice [22]. Especially in patients with severe mental illness and negative symptoms such as reduced drive and motivation, CRF serves as a control for the efficacy of an exercise intervention. Because of the above-mentioned low activity levels and high prevalence of cardiovascular comorbidities in patients with severe mental illness, besides aiming to improve symptoms of mental illness exercise interventions should also aim to increase CRF [91].

Several studies have focused on changes of CRF in patients with MDD [42, 45, 47–49, 84, 92–98] (Table 1) and SZ (e.g. [99–103]) (Table 2). Some studies directly measured maximal oxygen uptake (referred to as *V*O2max or *V*O2peak) to test changes in CRF in patients with MDD [45, 48, 49, 92, 95, 97, 98] and SZ [57, 63, 64, 85, 90, 99, 100, 103–106]. These tests are considered the gold standard, but other tests indirectly assessing CRF have been applied. For example, rather than being directly measured by cardiopulmonary exercise testing, *V*O2_max_ can be estimated by data from a maximal or submaximal stress test. This approach of estimating CRF was used in a few studies in MDD [42, 47, 84, 93] and one in SZ [61]. Submaximal proxy measures can also be used to estimate CRF, e.g., the 6-min walking test, 400-m walking test, and 3-min step test; some studies in SZ have used such tests [69, 101, 102, 107, 108]. Aerobic capacity or endurance capacity can also be measured by an exercise stress test without assessing oxygen uptake, an approach used in studies in MDD [96] and SZ [29, 58, 88].

Besides using different measurement methods, studies differ regarding the training modalities. To date, the effects of anaerobic exercise interventions have been investigated only scarcely. However, there are studies that combined aerobic and anaerobic training elements [90]. In addition, there is a growing body of studies investigating HIIT [33, 47, 98–100, 104], which is typically characterized by high-intensity exercise at 4 × 4 min intervals (85–95% of maximum heart rate [HR_max_]), with active breaks consisting of 3 min of moderate-intensity exercise (approximately 70% of HR_max_). HIIT was shown to be effective in improving CRF in patients with SZ. A small, 8-week study showed that *V*O2_peak_ increased by 12% in the HIIT group (*n* = 12) but did not increase in the PC gaming group (*n* = 7) [104]. These results were confirmed by a recent randomized controlled trial on the effects of 12 weeks’ HIIT on *V*O2_max_ in 21 patients with SZ. Like the study by Heggelund et al. [104], in this study the control group (*n* = 26) practiced their PC gaming skills. Although more than half of the patients in the HIIT group showed a significant increase in workload, a significant within-group difference in *V*O2_max_ was only observed when the physical activity competence of the health care providers was added into the statistical model. This result underlines the importance of professional and experienced supervision when aiming to successfully improve CRF in patients with SZ [99]. The study findings are supported by a similar study in which the training group (*n* = 25) performed aerobic interval training and received professional adherence support twice a week over the 12-week intervention period. The patients’ VO2peak improved by 10%, while no change was observed in the control group (*n* = 23), who performed two supervised exercise sessions at the beginning of the study and were subsequently encouraged to continue exercising on their own [100].

To the best of our knowledge, only two studies have evaluated a form of interval training in patients with MDD. Gerber et al. [98] found associations between an increase in *V*O2_max_ and improvements of symptoms in patients who performed a sprint interval training consisting of 25 repetitions of 30-s high-intensity bursts at 80% of maximal power output, followed by 30 s of total rest. Danielsson et al. [47] reported a significant increase of CRF in the intervention group, which performed intervals of exercise at higher perceived intensity during the aerobic exercise program, although training intensity was not strictly defined.

Exercise training of moderate intensity can also be effective in improving maximal oxygen consumption in patients with MDD [45, 92, 97] and SZ [29, 57, 63, 85, 105]. For example, in the most recent study in SZ, *V*O2_max_ improved by 18% in patients after a 12-week aerobic exercise program with intensities ranging from 60 to 75% of HR_max_ (*n* = 16) but decreased by − 0.5% in the usual care group (*n* = 17) [105].

Mixed programs consisting of aerobic training combined with resistance training may also have the potential to improve CRF in patients with MDD [49, 94] and SZ [85, 102]. Although three studies measured CRF directly by cardiopulmonary exercise testing [49, 85, 94, 106], Korman et al. [102] used a submaximal test (they assessed functional exercise capacity, a submaximal proxy measure of CRF, as the distance walked during the 6-min walking test). Moreover, two studies that evaluated the effect of dancing programs in SZ showed improvements in performance during the 6-min walking test [69] or the 3-min step test [107]. Overall, little evidence is available on the effects of mixed programs, so further studies are needed that use clearly defined exercise programs and high-quality CRF measurements.

In summary, in patients with severe mental illness aerobic exercise, especially endurance training, has shown beneficial effects on global functioning, cognition, and negative and depressive symptoms. It stimulates synaptic and brain plasticity and affects the volume of specific brain regions, with genetic risk (SZ-PRS) influencing the results. However, despite the growing body of literature, the type, duration, and frequency of exercise needed for beneficial effects in the long term have yet to be determined before aerobic exercise will be used widely in general practice [109]. Some recommendations for further studies can be given from the perspective of sports medicine:

- The American College of Sports Medicine recommends that adults engage in moderate-intensity cardiorespiratory exercise training for ≥ 30 min/day on ≥ 5 days/week for a total of ≥ 150 min/week, or vigorous-intensity cardiorespiratory exercise training for ≥ 20 min/day on ≥ 3 days/week for a total of ≥ 75 min/week [110].
- Further studies are needed to identify the most effective exercise interventions (type, duration, frequency).
- Because most studies were conducted over a relatively short intervention period of 3 to 4 months, were supervised and did not include a follow-up, future studies should focus on long-term adherence to exercise (e.g., by implementing motivational strategies supported by telemedicine and apps and by identifying and targeting typical barriers to exercise in this patient population).
- This patient group has a high prevalence of cardiovascular disease, so researchers should consider measuring the associated risk factors when performing exercise intervention studies.
- CRF should be comprehensively assessed in both clinical studies and clinical practice by direct measurements of maximal oxygen uptake.
- Exercise interventional studies in patients with BD need to be conducted because this patient group is underrepresented.
